# The Fami-life study: protocol of a prospective observational multicenter mixed study of psychological consequences of grieving relatives in French palliative care units on behalf of the family research in palliative care (F.R.I.P.C research network)

**DOI:** 10.1186/s12904-019-0496-4

**Published:** 2019-12-09

**Authors:** Maité Garrouste-Orgeas, Cécile Flahault, Edith Poulain, Adrien Evin, Frédéric Guirimand, Virginie Fossez-Diaz, Ségolène Perruchio, Catherine Verlaine, Anne Vanbésien, Willeme Kaczmarek, Laurence Birkui de Francqueville, Emmanuel De Larivière, Guillaume Bouquet, Laure Copel, Virginie Verliac, Véronique Marché, Carmen Mathias, Dominique Gracia, Alaa Mhalla, Véronique Michonneau-Gandon, Cécile Poupardin, Licia Touzet, Gaelle Ranchou, Virginie Guastella, Bruno Richard, Florent Bienfait, Marie Sonrier, Dominique Michel, Stéphane Ruckly, Sébastien Bailly, Jean-François Timsit

**Affiliations:** 1 IAME, INSERM, Université de Paris, F-75018 Paris, France; 2 Palliative Care unit, Reuilly Diaconesses Fondation, Rueil Malmaison, France; 3 Medical unit, French British Hospital, Levallois-Perret, France; 4 Service de médecine interne, Hôpital Franco Britannique, 4 rue Kléber, 92 300 Levallois-Perret, France; 5 Psychology laboratory and work process, Paris Descartes University, Paris, France; 6 Palliative Care unit, University Teaching Hospital, Nantes, France; 7 Palliative Care unit, Jeanne Garnier Institution, Paris, France; 8 UFR Simone VEIL – Santé, Versailles Saint Quentin en Yvelines University, Versailles, France; 9 Palliative Care unit, Bretonneau Hospital, Paris, France; 10 Palliative Care unit, Rives de Seine Hospital, Puteaux, France; 11 Palliative Care unit, General Hospital, Troyes, France; 12 Palliative Care unit, General Hospital, Douai, France; 13 Palliative Care unit, La Dracénie Hospital, Draguignan, France; 14 Palliative Care unit, Compiègne Noyon Hospital, Compiègne, France; 15 Palliative Care Unit, Marie Galène Institution, Bordeaux Caudéran, France; 16 Palliative Care unit, Tourcoing General Hospital, Tourcoing, France; 17 Palliative Care unit, Diaconesses Croix Saint Simon Hospital, Paris, France; 18 Palliative Care unit, Saintonge General Hospital, Saintes, France; 19 Palliative Care unit, Cognacq-Jay Hospital, Paris, France; 20 Palliative Care unit, Mulhouse Sud Alsace Hospital Network, Mulhouse, France; 21 Palliative Care unit, General Hospital, Salon-de-Provence, France; 22 Palliative Care unit, Albert Chenevier Hospital, Créteil, France; 23 Palliative Care unit, Castres-Mazamet General Hospital, Castres, France; 24 Palliative Care unit, General Hospital, Montfermeil, France; 25 Palliative Care unit, University Teaching Hospital, Lille, France; 26 Palliative Care unit, General Hospital, Périgueux, France; 27 Palliative Care unit, University Teaching Hospital, Clermont Ferrand, France; 28 Palliative Care unit, University Teaching Hospital, Montpellier, France; 29 Palliative Care unit, University Teaching Hospital, Angers, France; 30 Biostatistical department ICURESEARCH SA, Paris, France; 31 INSERM, CHU Grenoble Alpes, Grenoble Alpes University, HP2, Grenoble, France; 32 AP-HP, Bichat Hospital, Medical and infectious diseases ICU (MI2), F-75018 Paris, France

**Keywords:** Palliative care, Stress disorders, Post-traumatic, Relatives, Anxiety, Grief

## Abstract

**Background:**

Grieving relatives can suffer from numerous consequences like anxiety, depression, post-traumatic stress disorder (PTSD) symptoms, and prolonged grief. This study aims to assess the psychological consequences of grieving relatives after patients’ death in French palliative care units and their needs for support.

**Methods:**

This is a prospective observational multicenter mixed study. Relatives of adult patients with a neoplasia expected to be hospitalized more than 72 h in a palliative care unit for end-of-life issues will be included within 48 h after patient admission. End-of-life issues are defined by the physician at patient admission. Relatives who are not able to have a phone call at 6-months are excluded. The primary outcome is the incidence of prolonged grief reaction defined by an ICG (Inventory Complicate Grief) > 25 (0 best-76 worst) at 6 months after patient’ death. Prespecified secondary outcomes are the risk factors of prolonged grief, anxiety and depression symptoms between day 3 and day 5 and at 6 months after patients’ death based on an Hospital Anxiety and Depression score (range 0–42) > 8 for each subscale (minimal clinically important difference: 2.5), post-traumatic stress disorder symptoms 6 months after patient’ death based on the Impact of Events Scale questionnaire (0 best-88 worst) score > 22, experience of relatives during palliative care based on the Fami-Life questionnaire, specifically built for the study. Between 6 and 12 months after the patient’s death, a phone interview with relatives with prolonged grief reactions will be planned by a psychologist to understand the complex system of grief. It will be analyzed with the Interpretative Phenomenological Analysis. We planned to enroll 500 patients and their close relatives assuming a 25% prolonged grief rate and a 6-month follow-up available in 60% of relatives.

**Discussion:**

This study will be the first to report the psychological consequences of French relatives after a loss of a loved one in palliative care units. Evaluating relatives’ experiences can provide instrumental insights for means of improving support for relatives and evaluation of bereavement programs.

**Trial registration:**

NCT03748225 registered on 11/19/2018. Recruiting patients.

## Background

Bereavement of a loved one is an existential condition during which most people adequately adjust their emotions. However, in some cases, relatives can experience a psychological syndrome with various components such as symptoms of anxiety and depression, of post-traumatic stress-disorder (PTSD), of complicated or prolonged grief disorder (PGD), and increased mortality rates [1]. These conditions associate emotional, behavioral and cognitive symptoms (yearning, searching, detachment, numbness, bitterness, emptiness, and loss of sense and control). Quality of life might be jeopardized. It was initially termed complicated grief, then traumatic grief. In 2013, the classification of mental disorders (DSM-5) proposed to introduce a new diagnostic entity: « Persistent and Complex Bereavement-Related Disorder ». However, some researchers do not recognize it, insisting that evidence justifies the inclusion of PGD, but not complicated grief, as a new mental disorder [2]. The monitoring of bereaved relatives is an integral part of patient care in a patient-family centered care concept. Improving the relatives’ quality of life after a patient’s death is an international priority (Institute of Medicine or World Health Organization) [3–5].

Risk factors of prolonged grief disorders were reported in numerous studies [5–9]. Conflicts between relatives and health-care workers, poor communication and incomplete information during the palliative care stay [9], relatives characteristic’s: age, female gender [8, 10], pessimistic or anxious personality trait [10], financial issues [6], close relation-ship with the deceased patient [7, 10], absence of religiosity [10], and specific social context and family dynamics [11, 12] were reported as risk factors for prolonged grief. Numerous studies worldwide have evaluated post-loss emotional consequences. At 6 and 13 months, at least one psychological consequence was identified in 35.6 and 28.9%, respectively [13]. In Korea, anxiety and depression symptoms were reported for 58 and 57%, respectively [14]. In Australia, PTSD symptoms was diagnosed in 27.6 and 18.3%, respectively [13]. In Portugal, PGD was diagnosed in 28.6 and 15.6%, respectively [15]. In France, numerous studies are focused on population faced to end-of-life situations for intensive care patients [9, 16, 17]. Surprisingly, very few studies are available on relatives of palliative care patients. A French study reported depression in 25% of cancer patient’s spouses after a loss [18].

No data exists on the psychological experience of relatives having a relative hospitalized in palliative care or the incidence of prolonged grief in France. No specific recommendations are available for supporting grieving relatives.

Therefore, the focus of the study will be on prolonged grief reactions in relatives around 6 months after patient’s death in palliative care units. The methodology includes quantitative and qualitative data to better understand the complex, unique and intimate phenomena of grief and to identify needs for support.

## Methods

### Study design

The Fami-Life study is a prospective observational multicenter mixed study to assess the psychological consequences of a loss of a loved one in palliative care units. We designed a quantitative study to assess the psychological consequences of relatives 6 months after patients’ death and a qualitative study to examine the prolonged grieving process. The study timeline and schedule of enrollment, interventions and assessments are presented in Table 1.

**Table 1:** Study timeline, schedule of enrollment, intervention and assessments

	Enrollment		Follow-up		
Time points	Within 48 h after Palliative unit admission		Between day 3 and 5 after palliative unit admission	At 6 months	Between 6-months and 1-year
Enrollment					
Eligibility screen	X				
Inform consent	X				
Inclusion	X				
Assessment					
HADS of relatives			X		
(random order)					
IES-R of relatives				X	
HADS relatives				X	
ICG				X	
FAMI-LIFE questionnaire				X	
Qualitative study (interview of relatives with ICG > 25)					X

### Participants

#### Recruitment of palliative care units

The participating palliative care units belong to the French Society of Palliative Care (SFAP) and are selected using the units list on its website (Table 2). An invitation of participation is sent to the clinical responsible of each unit.

**Table 2:** Participating Palliative Care centers

Guillaume Bouquet, MD	Centre Hospitalier, Tourcoing
Laurence Birkui de Franqueville, MD	Centre Hospitalier Compiègne Noyon, Compiègne
Florent Bienfait, MD	Centre Hospitalier Universitaire, Angers
Laure Copel, MD	Diaconesses Croix Saint Simon Hospital, Paris
Emmanuel Delarivière, MD	Maison Marie Galène, Bordeaux
Adrien Evin, MD	Centre Hospitalier Universitaire, Nantes
Virginie Fossez-Diaz, MD	Hôpital Bretonneau, Paris
Maité Garrouste-Orgeas, MD	Fondation Diaconesses Reuilly, Rueil Malmaison
Virginie Guestella	Centre Hospitalier universitaire, Clermont Ferrand
Frédéric Guirimand, MD, PhD	Maison médicale Jeanne Garnier, Paris
Dominique Gracia, MD	Centre Hospitalier, Salon de Provence
Willeme Kaczmarek, MD	Centre Hospitalier de la Dracénie, Draguignan
Alaa Mhala, MD, PhD	Hôpital Albert Chenevier, Créteil
Véronique Marché, MD	Fondation Cognac-Jay, Paris
Carmen Mathias, MD	GHR Mulhouse Sud Alsace, Mulhouse
Véronique Michonneau-Gandon, MD	Centre Hospitalier Inter-Communal Castres Mazamet
Ségolène Perruchio, MD	Centre Hospitalier Rives de Seine, Puteaux
Edith Poulain, MD	Maison médicale Notre Dame du Lac, Rueil Malmaison
Cécile Poupardin, MD	Centre Hospitalier Intercommunal Raincy-Montfermeil, Montfermeil
Gaelle Ranchou, MD	Centre Hospitalier, Périgueux
Bruno Richard, MD, PhD	Centre Hospitalier Universitaire Montpellier
Licia Touzet, MD	Centre Hospitalier Universitaire, Lille
Anne Vanbésien, MD	Centre Hospitalier, Douai
Catherine Verlaine, MD	Centre Hospitalier, Troyes
Virginie Verliac, MD	Centre Hospitalier de Saintonge, Saintes

#### Recruitment of patients and relatives

##### Inclusion criteria

All the inclusion criteria must be fullfilled within 48 h after patient admission:

Patients with a neoplasia should be hospitalized more than 72 h for end-of-life issues. End of life issues are assessed after the first evaluation by the palliative care physician.

Relatives are susceptible to visit the patient during their stay. Relatives are defined as spouse, partner, children, siblings, parents and cousins and any relatives with a strong attachment link. The study will be proposed to relatives present during the first meeting and within 48 h after patient admission. All relatives present can be included because the patient’s death affects the entire group of relatives and not a single individual.

##### Non inclusion criteria

- Patients under any form of legal guardianship
- Patients with less than 72 h of life-expectancy
- Patients or relatives who refused the study
- Patients or relatives included in a study using the same questionnaires
- Patients without relatives visiting them within the first 48 h after admission
- Patients not hospitalized for end-of-life issues
- Relatives not fluent in French
- Deaf or mute relatives

#### Data collection

##### Center characteristics

All data will be collected on an e-CRF built by the ICUREsearch company (Contract Research Organization). Data will be on center, patients and relatives. Palliative care unit characteristics are the following: hospital type (university, community, or private hospital) number of palliative care beds, number of senior physicians and fellows, physician-to-patient ratio, nurse-to-patient ratio (day and night), presence of a waiting room and relative room, availability of a psychologist, physiotherapist, social worker, occupational and music therapist, biographer; availability of interpret or religious services; availability of a dedicated nurse for relative meetings, of volunteers, of speaking group for relatives; time intervals allowed for visit; organization of different meetings (admission, weekly, death period), delivery of an information leaflet at admission and after death, delivery of a children leaflet, delivery of a condolence letter, bereavement follow-up process, availability of a 24-h visitation policy, age for children visitation, possibility for a pet to visit the patient, availability of staying during the night (bed in the patient ‘room, dedicated room, other), and participation in patient care.

##### Patient’s characteristics

The following data will be collected: demographic characteristics, diagnosis and localization of the neoplasia, presence of metastases, diagnosis of comorbid conditions, symptoms present on admission, presence of advances directives and of a surrogate, patient’s status at discharge, and length of stay. Request of euthanasia at admission or during the stay by patient and use of a continuous sedation until death will be collected.

##### Relatives’ characteristics

The following data will be collected: demographic characteristics, relationship with the patient, educational level, professional occupancy, whether they already have a next one previously hospitalized in a palliative care unit, whether they are the surrogate of the patient, and the presence of health workers at home before admission (nurse, nursing-assistant or occupational worker). The use of “hospital at home” services will also be collected. Request of euthanasia at admission and during the stay by relatives will be collected.

#### Organization of the study

In each center, a local team (physician and nurse or psychologist) will conduct the study. Consecutive patients will be approached within the first 48 h after palliative care admission. Examination of eligibility, its confirmation and inclusion will be performed by the local team. If the relative agrees, the patient will be invited to participate in the study giving permission of using his/her baseline medical information. After inclusion, between day 3 and day 5 after patient admission, a HADS questionnaire [19] will be administered to each relative included in the study.

At 6 months after the patient’s death, the psychologist will call the included relatives to fulfil the questionnaires in a random order (HADS [19], PTSD [20], IGC [21, 22]. The Fami-Life questionnaire, specifically built for the study, will report 12 questions that assess the information, communication, support and grief interventions (Table 3). The assessment will be on a Likert scale for the first three questions. All other questions will be explored with a Yes/No answer. The satisfactory questionnaire (Fami-Life) evaluating the last moments of life will be given in the last position because it may require more listening time. If we cannot reach a relative after 5 phone calls, he/she will be considered as lost-to-follow up. Only relatives of patients deceased in palliative care unit will be called.

**Table 3:** Questions of the Fami-Life questionnaire

1. Have you been satisfied with the information received in the palliative care service?	
2. Were you satisfied with the support your loved one received?	
3. Were you satisfied with the care that health-care workers offered you?	
4. Have you been warned of the aggravation of your loved one?	
5. Did you meet a doctor at the time of the death?	
6. Were you in the room at the time of the death?	
7. Have you received a letter of condolence from the team?	
Would you have liked to receive one?	
8. Were you offered a follow-up of bereavement?	
9. Since the death of your loved one, are you taking drugs that you did not take before?	
If yes, which drugs?	
10. Are you followed by a psychologist?	
If yes: from the ward, or other	
11. Do you participate in a speaking group with other relatives or relatives in the same situation as you?	
12. Would have you liked to have a follow-up after the death of your loved one?	

Between 6 and 12 months, the psychologist will call the relatives previously selected (those with an IGC > 25) for a telephone interview assessing their grieving process.

#### Study management

The steering committee comprises three palliative care physicians (MGO, DM, LC), three psychologists (CF, LF, MS), a biostatistician (SR) and two methodologists (SB, JFT). MGO and DM will select and open the centers. MGO, LC, DM are in charge of addressing any questions of the investigators and checked for potential inconsistencies. CF, LF and MS are responsible of the qualitative study, will interview the relatives with prolonged grief and perform the qualitative analysis. The local investigators (physician and nurse) are responsible of the inclusion of patients and relatives, of collecting the signed informed consents, of the acquisition of all data of the study, of fulfilling the e-CRF, and of sending all the documents required for the follow-up by the psychologist. Each local investigator is accountable for the accuracy in ensuring that any part of the work and related-questions is appropriately resolved.

### Outcomes

#### Primary outcome

The primary outcome is to assess the incidence of prolonged grief of relatives 6 months after the patient’s death in a palliative care unit.

#### Measure of the primary outcome

The intensity of grief reactions is evaluated with the French validated version of the IGC (Inventory of Complicated Grief) [21–23], the most commonly used assessment tool for complicated grief. It was developed by PRIGERSON et al [23] and focuses on symptoms that are distinguishable from symptoms of depression and anxiety. Moreover, the ICG was designed to distinguish between normal reactions and more pathological forms of grief. It consists of 19 first-person statements (such as “Ever since he/she died, it is hard for me to trust people”). Each item is rated from 0 (not at all) to 4 (severe). We defined complicated grief with an IGC scores > 25 (0 best-76 worst). No published minimal clinically difference was reported for the IGC.

#### Prespecified secondary outcomes

1. Anxiety and depression symptoms in relatives between day 3 and day 5 after patient admission in a palliative care unit
2. Anxiety and depression symptoms in relatives 6 months after patient’s death
3. Post-traumatic stress disorder symptoms in relatives 6 months after patient’s death
4. Risk factors for prolonged grief
5. Report of the relative’s grief experience in those who have signs of prolonged grief

#### Measure of the prespecified secondary outcomes

Prespecified secondary outcomes (anxiety, depression, post-traumatic stress disorder symptoms and exploration of relatives’ experience during hospitalization) will be explored during a telephone interview performed by a psychologist specifically used for approaching grieving relatives.

Anxiety and depression syndrome will be evaluated based on The Hospital Anxiety and Depression syndrome (HADS) [19] score, range 0 (best)- 42 (worst). The HADS is valid and reliable, easy to administer, and has been successfully used to measure symptoms of anxiety (HADS-subscale Anxiety) and depression (HADS-subscale Depression) in relatives of palliative care patients [24]. Significant anxiety and depression symptoms will be defined by a score > 8 for anxiety and depression subscales. The minimal clinically important difference is 2.5 for each subscale [25].

Post-traumatic stress disorder symptoms will be evaluated in relatives at 6 months as measured by the Impact Event Scale-Revised questionnaire (IES-R). The IES-R is a valid and reliable scale that has been used successfully in family members of palliative care patients [26]. The instrument contains subscale items on intrusion, avoidance and hyperarousal. We define significant post-traumatic stress disorder symptoms by an IES-R score > 22 (range 0 best-88 worst) [27, 28]. We did not identify any published definition of the minimal clinically important difference for the IES-R on the 0–88 scale.

Risk factors of prolonged grief will be explored through the center, patient and family characteristics. Exploration of relative’s experience during the palliative care hospitalization will be assessed with the FAMI-LIFE questionnaire (12 questions), specifically built for the study by the scientific committee. It reports data about communication features between healthcare clinicians and relatives, information about the last moments of life, and relatives’ needs after death.

Relative grief experience. Experience of grief will be evaluated between 6 and 12 months after patient’ death. A purposive subset of relatives with heterogeneous characteristics and with prolonged grief will be included. Interviews that will be conducted will target to describe the subjective experience of grief among the relatives, and more specifically their meaning-making process. A psychologist will conduct the interviews through phone calls. All phone calls will be centralized. Each interview will be audio-recorded, and the full transcript will be subjected to an Interpretative Phenomenological Analysis (IPA) [29] that will be performed by CF, LF and MS. IPA is designed to understand the complex system of meanings attached to a unique, subjective and eminently intimate phenomenon [18, 30]. It seems the most appropriate method to investigate the experience of losing a loved one and to identify needs for support. IPA aims to offer insights into how a given person, in a given context, makes sense of a given phenomenon. Usually these phenomena relate to experiences of some personal significance, such as a major life event.

### Sample size justification

Prolonged grief reactions were reported in 24.4% [10] at 8 months, and 25.4% [31], 28.6% [15], 30% [32], and 40% [33] at 6 months. We anticipated an incidence of prolonged grief of 25% 6 months after patient death. Horowitz et al reported that the frequency of grief symptoms declined within 6 to 12 months after the bereavement [34]. Grief reactions decreased as a function of time from death with a steepest decrease from the first month to the sixth month after death, and without any significant decrease from 6 months to 13 months [33, 35]. For the current study, we selected a time period of 6 months after the death. According to the medical knowledge, longitudinal studies of relatives at 6 months usually report a participation rate around 60%. To detect the risk factors associated with a prolonged grief with an odd ratio of 2, an alpha risk of 0.05 and a power of 0.80, we anticipate that 500 decedents and their relatives are necessary (assuming the independence of prolonged grief in the relatives of the same family).

### Statistical analysis plan

Patient, relatives and center-variables are reported as numbers (percentage) for qualitative data and medians (Q1–Q3) for quantitative data. For continuous variables, we perform tests of logit linearity and transformation if necessary. Strategy of imputation of missing values will be adapted according to the pattern of missing values. The missing data will be considered by a simple imputation method in case of low frequency (less than 2%) or by multiple imputation if the frequency is higher. Variables with more than 30% missing data will not be included in the analysis. No imputations will be performed for patient outcomes. The primary outcome is the incidence of relatives with an IGC score > 25. The incidence rate will be defined by the ratio between the number of persons who have a prolonged grief on the number of person-time in the study. A 95% Wald confidence interval will be computed for the incidence rate.

To assess the risk factors for prolonged grief in patients’ relatives, multivariable hierarchical logistic regression model will be performed, with a random effect on the center. An univariable analysis will be first performed to select the variables associated to the outcome with a *p*-value threshold of 0.2 to account for the main confounding factors. The relative effect can be considered either as a random effect or a fixed effect in the model. We will then perform multivariable logistic or negative-binomial models for the modification of anxiety, depression and PTSD scores for the secondary criteria. To account for the rate of lost-to-follow-up within the relatives, a weighting on the inverse probability of censoring weight (IPCW) will be performed. All statistical analyses were performed using the SAS software (SAS 9.4, SAS Institute, Cary, NC). A *p* value < 0.05 will be considered significant.

To describe the experience of bereaved relatives through an interview, a purposive subset of relatives will be selected. We will comply with the recommendations regarding purposive sample in Interpretative Phenomenological Analysis [29]. This sample will be purposive with heterogenous characteristics. According to the IPA guideline, participation of at least 15 people will allow a broad and relevant understanding of the meaning-making process of the grief experience. To maximize the meaning making heterogeneity, we plan to recruit women and men, from various palliative units, from various geographical areas, of various ages, and with various types of relationship with the deceased. We will stop the recruitment when the adequate variety of meaning making processes will be obtained.

## Discussion

This study will depict the grieving process of relatives after patient ‘s death in palliative care in France. According to the World Health Organization definition of palliative care, providing support for the relatives during their grieving process is an inherent part of palliative care services [36]. Currently, there is no consensus to which type and amount of support is indicated to meet the demand of the WHO definition of bereavement support in palliative care [37]. There are hardly substantial pieces of knowledge on how bereavement of relatives of palliative care patients is handled. The need for support in the bereavement period was poorly predicted by the healthcare clinicians [33]. Having baseline data of the incidence rate of prolonged grief and other psychological consequences, of risk factors of relative’s prolonged grief, and better knowledge of the management of end-of-life issues in palliative care units will increase our knowledges on the grieving process. Currently, palliative care team have different strategies for managing grieving relatives. No scientific data are available on these practices, and there are no national guidelines with a sufficient high grade of proofs.

The strengths of this study will be the high number of relatives included, and the multicenter design with the recruitment of patients from a large set of centers with patient’s characteristics consistent with those of patients usually admitted in palliative care in France, which will ensure that results could be generalized. The mixed design will enable to approach how relatives experience the period of bereavement and to help healthcare clinicians in targeting interventions to decrease bereaved relative’s burden.

This study will have several limitations. First, the exploration of psychological consequences will focus on patients with neoplasia, who do not represent all the case-mix of patients admitted in palliative cares. Second, evaluation will be performed at only one time-point, which is insufficient to describe the evolution of the grieving process. We choose one point to limit the rate of lost-to-follow-up among relatives. Third, the centers are all in France, and the study results might not be generalized to other countries.

Our study is part of a scientific approach to the management of grief in palliative care to build a scientifically valid management program.

## Data Availability

No applicable.

## Abbreviations

CNIL: Commission Nationale de l'Informatique et des Libertés
DSM-5: Standard Classification of Mental disorders
FRIPC: Family Research in Palliative Care
HADS: Hospital Anxiety and Depression Scale
ICG: Inventory of Complicated Grief
IES-R: Impact of Event Scale-Revised
IPA: Interpretative Phenomenological Analysis
PGD: Prolonged Grief Disorder
PTSD: Post-traumatic stress disorder
SFAP: French society of Palliative Care
